# Number of Pretransplant Therapeutic Plasma Exchange Sessions Increase the Recurrence Risk of Hepatocellular Carcinoma in ABO-Incompatible Living Donor Liver Transplantation

**DOI:** 10.3389/ti.2025.14304

**Published:** 2025-08-13

**Authors:** Young Jin Yoo, Deok-Gie Kim, Eun-Ki Min, Seung Hyuk Yim, Mun Chae Choi, Hwa-Hee Koh, Minyu Kang, Jae Geun Lee, Myoung Soo Kim, Dong Jin Joo

**Affiliations:** ^1^ Department of Surgery, Research Institute for Transplantation, Yonsei University College of Medicine, Seoul, Republic of Korea; ^2^ Division of Transplant Surgery, Department of Surgery, Yongin Severance Hospital, Yonsei University College of Medicine, Seoul, Republic of Korea; ^3^ Department of Surgery, Graduate School of Medicine, Yonsei University College of Medicine, Seoul, Republic of Korea

**Keywords:** ABO-incompatible living donor liver transplantation, hepatocellular carcinoma, plasma exchange, surgical oncology, oncologic outcome

## Abstract

Previous studies have reported comparable oncologic outcome between ABO-incompatible (ABOi) living donor liver transplantation (LDLT) and ABO-compatible (ABOc) LDLT in patients with hepatocellular carcinoma (HCC). We aimed to analyze the relationship between number of therapeutic plasma exchanges (TPE) before LDLT and HCC outcomes in ABOi LDLT. In this single-center retrospective study, 428 adult LDLT recipients with HCC were categorized into three groups according to ABO incompatibility and the number of pretransplant TPE: ABOc (n = 323), ABOi/TPE ≤5 (n = 75), and ABOi/TPE ≥6 (n = 30). The RFS and HCC recurrence rates were compared. Three groups showed similar characteristics in most demographics, pretransplant tumor markers and pathologies. The median initial isoagglutinin (IA) titer was 1:64 (range negative-1:512) in ABOi/TPE ≤5 group and 1:512 (range 1:128–1:4,096) in ABOi/TPE ≥6 group. Five-year RFS was significantly lower (75.7% vs. 72.7% vs. 50.0%, P = 0.005) and HCC recurrence was significantly higher in the ABOi/TPE ≥6 group than in the other groups(16.4% vs. 17.0% vs. 39.4%, P = 0.014). In multivariable Cox regression analysis, ABOi/TPE ≥6 was an independent risk factor for RFS (aHR 1.99, 95% CI:1.02–3.86, P = 0.042) and HCC recurrence (aHR 2.42, 95% CI:1.05–5.57, P = 0.037). More than six pretransplant TPE sessions may increase the risk of HCC recurrence after ABOi LDLT. Reducing TPE sessions to fewer than six should be considered while maintaining immunological stability through IA titer control.

## Graphical Abstract

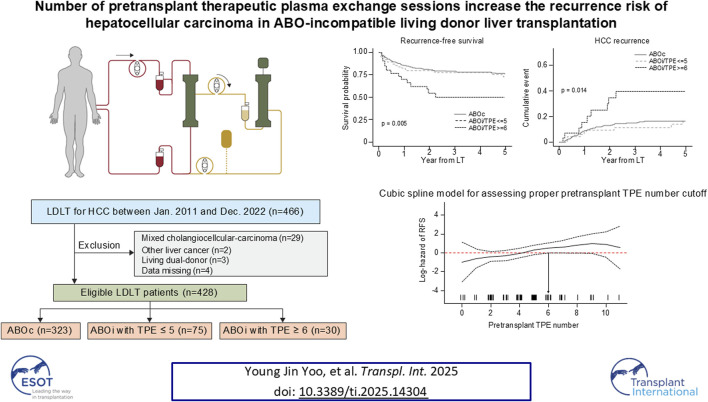

## Introduction

Liver transplantation (LT) is an effective, and sometimes the only, treatment option for unresectable hepatocellular carcinoma (HCC). However, owing to organ shortages, not all patients can receive timely LT. Consequently, the demand for living donor liver transplantation (LDLT) for HCC is increasing worldwide, and numerous studies have reported comparable oncological outcomes between LDLT and deceased donor liver transplantation (DDLT) [1–7].

When an ABO-incompatible (ABOi) living donor is the only available option, ABO-incompatible LDLT (ABOi LDLT) with proper desensitization becomes a viable choice [8–15]. Despite the need for pretransplant antibody treatment and an increased risk of posttransplant infections, ABOi LDLT has been reported as a feasible treatment for patients with end-stage liver disease, offering substantial survival benefits even for those with high Model for End-Stage Liver Disease (MELD)scores [11, 12, 16, 17]. Additionally, several Korean centers have reported that ABOi LDLT has a similar impact on HCC outcomes compared to ABO-compatible (ABOc) LDLT (ABOc LDLT) [12, 18–21].

Despite these reports, ABOi LDLT necessitates more potent immunosuppression, including B-cell depleting agents, therapeutic plasma exchange (TPE), and higher maintenance immunosuppressants, which raises concerns about potentially adverse oncologic outcomes [22, 23]. Furthermore, ABOi LDLT requires additional pretransplant TPE sessions as the titer of blood group antibodies increases. However, there are no published studies examining the differences in HCC outcomes based on the degree of desensitization required.

Therefore, this study aimed to analyze the effect of the number of pretransplant TPE sessions, a critical component of pretransplant treatment, on HCC outcomes in ABOi LDLT.

## Materials and Methods

### Study Material

In this retrospective cohort study, we analyzed single-center data from 466 patients who underwent LDLT for HCC between January 2011, when ABOi LDLT was initiated at our institution, and December 2022. The baseline characteristics and details of explant pathology were retrieved from a prospectively collected institutional database. The exclusion criteria were as follows: mixed cholangiocellular carcinoma on pathology (n = 29), liver cancer other than HCC (n = 2), LDLT from a dual living donor (n = 3), and missing data (n = 4) (Supplementary Figure S1, study population).

A total of 428 eligible patients were categorized according to ABO incompatibility and the number of pretransplant TPE sessions: ABO-compatible (ABOc group, n = 323, 75.5%), ABO-incompatible with fewer than 5 TPE sessions (ABOi/TPE ≤5 group, n = 75, 17.5%), and ABO-incompatible with six or more TPE sessions (ABOi/TPE ≥6 group, n = 30, 7.5%). The cutoff for the number of TPE sessions (6 times) was determined based on the spline curve for recurrence-free survival (RFS), where the hazard began to significantly increase (Supplementary Figure S2, spline curve).

### Data Collection and Outcomes

All relevant information regarding recipients, donors, and LDLT surgery was retrieved from the institutional database. The underlying liver diseases associated with HCC included hepatitis B, hepatitis C, and non-B/non-C. Detailed information on explant pathology and tumor markers, such as alpha-fetoprotein (AFP) and protein induced by vitamin K absence or antagonist-II (PIVKA-II) at the time of LDLT, was obtained. Additionally, data on pretransplant locoregional and systemic treatments, as well as previous hepatectomies, were collected for patients with HCC. RFS and HCC recurrence (time to recurrence) were the primary outcomes.

### Pretransplant Desensitization for ABO Incompatibility

Our institutional protocol for desensitization in ABOi LDLT mainly consisted of rituximab and TPE, as described previously [24, 25]. Every pretransplant TPE sessions and desensitization protocols were performed within 2 weeks prior to ABOi LDLT. A recently revised version of this protocol is provided in Supplementary Figures S3-1–S3-2 (Desensitization protocol for ABOi LDLT). For the initial and target isoagglutinin (IA) titers, higher IgM or IgG anti-A/B titers were employed. The number of preoperative TPE sessions was determined based on the initial IA titer, the response to TPE, and the decrease in the ABO titer. Splenectomy and postoperative TPE were performed in patients at high risk of rejection, specifically those with an IA titer greater than 1:64 at the time of LT. Additional rounds of TPE were conducted postoperatively in cases of clinical rejection or IA titer rebound, defined as a resurgence to 1:64 and a minimum two-fold increase. Following TPE, intravenous immunoglobulin (IVIG) was administered at a dose of 500–800 mg/kg on an individualized basis, depending on ABO antibody levels and infection risk.

### Statistical Analysis

Depending on the type of variable, data are presented either as numbers (percentages) or as medians (interquartile range [IQR]). The Mann–Whitney U test or chi-square test was employed to compare continuous and categorical variables, respectively, when appropriate. HCC outcomes were analyzed using Kaplan–Meier curves and log-rank tests. Multivariable Cox regression was performed to evaluate HCC outcomes in the entire cohort, including covariates with significant P values <0.1 from the univariable analysis. In the risk analysis of HCC recurrence, non-HCC death was considered a competing risk, utilizing the Fine and Gray method [26] for competing risk regression. In the ABOi LDLT groups, the 5-year estimates of HCC recurrence were compared based on the number of TPE sessions (≤5 vs. ≥6) across various subgroups categorized by tumor burden, which reflects the tumor size, tumor number, and AFP and PIVKA-II levels [27–30], as well as ABO antibody strength, postoperative rebound of IA titer and TPE, and splenectomy status. Subgroup analyses were conducted in a univariate manner due to the small size of each group. All statistical analyses were performed using the R statistical package, version 4.3.0 for macOS^1^, with the significance threshold set at P < 0.05.

### Statement of Ethics

This study was performed in accordance with the Declaration of Helsinki and the Declaration of Istanbul and was approved by the Institutional Review Board of Severance Hospital, Yonsei University Health System (IRB number 4-2024-0977). The requirement of informed consent was waived due to the retrospective nature of the study.

## Results

### Baseline Characteristics

No significant difference was noted in most baseline patient characteristics (Table 1). The distribution of LT years was also not statistically significant (P = 0.069); however, a higher proportion of transplants in the ABOi groups occurred between 2016 and 2019. Most patients had hepatitis B as the underlying cause of HCC across all groups, with no statistical significance (76.8% in ABOc, 69.3% in ABOi/TPE ≤5, and 86.7% in ABOi/TPE ≥6, P = 0.401). Notably, the ABOi/TPE ≥6 group required a significantly higher number of red blood cell transfusions (median 4.5 packs) than the ABOc and ABOi/TPE ≤5 groups (median two packs, P = 0.014). No significant differences were noted in the pretransplant AFP and PIVKA-II levels. Additionally, history of hepatectomy, locoregional therapy (LRT), and systemic treatment were similar across the groups.

**TABLE 1:** Baseline characteristics of patients, according to ABO incompatibility and the number of pretransplant therapeutic plasma exchange.

Variables	ABOc (n = 323)	ABOi/TPE ≤5 (n = 75)	ABOi/TPE ≥6 (n = 30)	*P*
Age, years	56.8 ± 7.0	57.1 ± 6.9	55.6 ± 7.3	0.608
Sex, female	58 (18.0)	17 (22.7)	7 (23.3)	0.539
BMI	23.8 (22.3–26.1)	24.9 (23.4–26.3)	24.0 (21.9–25.9)	0.065
LT year				0.069
2011–2015	112 (34.7)	15 (20.0)	7 (23.3)	
2016–2019	104 (32.2)	33 (44.0)	14 (46.7)	
2020–2022	107 (33.1)	27 (36.0)	9 (30.0)	
Underlying liver disease for HCC				0.401
Hepatitis B	248 (76.8)	52 (69.3)	26 (86.7)	
Hepatitis C	23 (7.1)	8 (10.7)	1 (3.3)	
Non-B, Non-C	52 (16.1)	15 (20.0)	3 (10.0)	
Hypertension	74 (22.9)	21 (28.0)	9 (30.0)	0.490
Diabetes mellitus	97 (30.0)	27 (36.0)	12 (40.0)	0.367
Pretransplant MELD	10 (8–14)	10 (8–13)	11.5 (8–14)	0.674
Donor age, years	31 (24–40)	34 (26–40.5)	35 (25–41)	0.203
Donor sex, female	130 (40.2)	26 (34.7)	11 (36.7)	0.647
GRWR^a^ <0.8	27 (8.4)	5 (6.7)	2 (6.7)	0.856
Macrovesicular steatosis ≥10%	46 (15.2)	9 (12.3)	2 (6.9)	0.422
Cold ischemic time, min	126 (106–150)	126 (102–152.5)	128.5 (96–180)	0.884
Transfusion RBC, packs	2 (0–6)	2 (0–7.5)	4.5 (2–9)	0.014
AFP at LT, ng/mL	6.6 (3.3–23.1)	6.4 (3.3–14.0)	4.3 (2.2–27.2)	0.545
PIVKA at LT, mAU/mL	38 (22–112)	38 (23.5–141)	47 (20–232)	0.666
Hepatectomy history	62 (19.2)	13 (17.3)	7 (23.3)	0.779
Pretransplant LRT	246 (76.2)	59 (78.7)	23 (76.7)	0.899
Systemic treatment	45 (13.9)	9 (12.0)	5 (16.7)	0.812
Explant pathology				
Total necrosis	57 (17.6)	13 (17.3)	4 (13.3)	0.836
Viable tumor number	1 (1–3)	2 (1–3)	2 (1–3)	0.485
Maximum tumor size, cm	1.7 (1.0–3.0)	1.8 (0.8–3.0)	2.4 (1.3–3.7)	0.139
Microvascular invasion	76 (23.5)	20 (26.7)	9 (30.0)	0.656
Poor differentiation	107 (33.1)	22 (29.3)	13 (43.3)	0.388
Satellite nodule	35 (10.8)	8 (10.7)	8 (26.7)	0.035
PVTT	5 (1.5)	2 (2.7)	1 (3.3)	0.673

Results presented as number (percentage) or median (interquartile range) values.

^a^ Graft weight was directly measured during operation.

ABOc, ABO, compatible; ABOi, ABO incompatible; AFP, alpha-feto protein; BMI, body mass index; GRWR, graft recipient weight ratio; HCC, hepatocellular carcinoma; LRT, locoregional treatment; LT, liver transplantation; MELD, model for end-stage liver disease; PIVKA, protein induced by vitamin K antagonist-II; PVTT, portal vein tumor thrombosis; TPE, therapeutic plasma exchange.

Most characteristics from explant pathology were similar across the groups, including the incidence of portal vein tumor thrombosis (PVTT), total necrosis, number of viable tumors, maximum tumor size, microvascular invasion, and poor differentiation. However, the presence of satellite nodules was significantly higher in the ABOi/TPE ≥6 group (26.7%) than in the other groups (10.8% in ABOc and 10.7% in the ABOi/TPE ≤5, P = 0.035).

### Detailed Information on Recipient of ABOi LDLT

Almost all patients who underwent ABOi LDLT received rituximab and at least one cycle of TPE for desensitization. Table 2 presents details regarding ABO incompatibility and desensitization protocols for patients in the ABOi group, categorized by the number of pretransplant TPE sessions. A significantly higher proportion of A to O transplants was observed in the ABOi/TPE ≥6 group (56.7%) than in the ABOi/TPE ≤5 group (17.3%, P < 0.001). The median IA titer was significantly higher in the ABOi/TPE ≥6 group than in the ABOi/TPE ≤5 group at initial assessment (1:64 vs. 1:512, P < 0.001), at the time of LT (1:8 vs. 1:32, P < 0.001), and after LT (1:16 vs. 1:32, P < 0.001).

**TABLE 2:** Details for ABO incompatibility and desensitization of ABO incompatible group patients, according to therapeutic plasma exchange number.

Variables	ABOi/TPE ≤5 (n = 75)	ABOi/TPE ≥6 (n = 30)	*P*
ABO type			<0.001
*A*	36 (48.0)	2 (6.7)	
*B*	16 (21.3)	1 (3.3)	
*O*	23 (30.7)	27 (90.0)	
Donor ABO type			0.008
*A*	23 (30.7)	17 (56.7)	
*AB*	25 (33.3)	2 (6.7)	
*B*	27 (36.0)	11 (36.7)	
A to O	13 (17.3)	17 (56.7)	<0.001
IA titer at initial	1:64 (1:24–1:128)	1:512 (1:256–1:1024)	<0.001
IA titer at LT	1:8 (1:4–1:16)	1:32 (1:16–1:64)	<0.001
Pretransplant TPE number	3 (2–4)	6.5 (6–7)	<0.001
Pretransplant IVIG^a^	8 (10.7)	16 (53.3)	<0.001
Rituximab	73 (97.3)	30 (100.0)	0.910
Rituximab conventional dose^b^	54 (72.0)	26 (86.7)	0.078
Pretransplant duration of MMF	7 (4–8)	7 (0–8)	0.645
Pretransplant MMF total dose, mg	3,500 (3,000–4,000)	3,500 (2000–4,000)	0.284
Splenectomy	4 (5.3)	7 (23.3)	0.018
Posttransplant IA titer rebound^c^	19 (25.3)	8 (26.7)	0.986
Posttransplant maximum IA titer	1:16 (1:4–1:48)	1:32 (1:16–1:128)	0.001
Posttransplant TPE^d^	14 (18.7)	11 (36.7)	0.049
Posttransplant IVIG^a^	6 (8.0)	9 (30.0)	0.009

Results presented as number (percentage) or median (interquartile range) values.

^a^ Pretransplant IVIG total dose range was 7.5–50 g in ABOi/TPE ≤5 group, and 5.5–136 g in ABOi/TPE ≥6 group. Posttransplant IVIG total dose range was 15–127.5 g in ABOi/TPE ≤5 group, and 1–458 g in ABOi/TPE ≥6 group.

^b^ 375 ± 25 mg per body surface area (m2).

^c^ Defined as IA titer increased to more than 1:64 after transplantation.

^d^ Posttransplant TPE number ranges 0–10 in ABOi/TPE ≤5 group, and 0–15 in ABOi/TPE ≥6 group.

ABOi, ABO incompatible; IA, isoagglutinin; IVIG, intravenous immunoglobulin; LT, liver transplantation; MMF, mycophenolate mofetil; TPE, therapeutic plasma exchange.

Additionally, a significantly higher proportion of patients in the ABOi/TPE ≥6 group underwent splenectomy (5.3% vs. 23.3%, P = 0.018), pretransplant IVIG (10.7% vs. 53.3%, P < 0.001), posttransplant IVIG (8.0% vs. 30.0%, P = 0.009), and posttransplant TPE (18.7% vs. 36.7%, P = 0.049). The univariate analysis showed no significant association between recipient or donor ABO blood type and 5-year HCC recurrence, regardless of TPE sessions. Similarly, A to O donor-recipient mismatches did not show a significant impact on recurrence risk (Supplementary Table S1).

### HCC Outcomes

**FIGURE 1:**
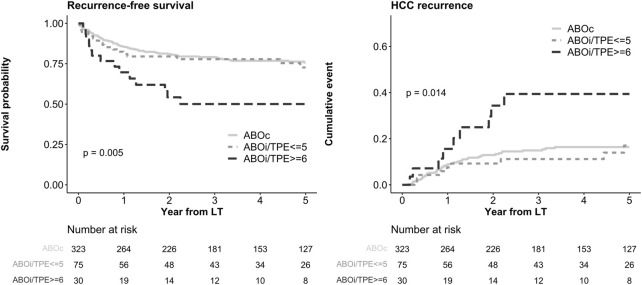
Kaplan-Meier curve of RFS and HCC recurrence according to ABO incompatibility and plasma exchange numbers. RFS, recurrence free survival; HCC, hepatocellular carcinoma; ABOc, ABO compatible; ABOi, ABO incompatible; TPE, therapeutic plasma exchange; LT, liver transplantation.

As shown in the Kaplan-Meier curves in Figure 1, a significant difference was observed in RFS between the ABOc group and the ABOi/TPE ≥6 group (5-year survival: 75.7% in the ABOc group vs. 50.0% in the ABOi/TPE ≥6 group, P = 0.005). Additionally, the HCC recurrence rates also differed significantly (5-year survival: 16.4% vs. 39.4%, P = 0.014).

To further evaluate the impact of TPE on oncologic outcomes, we categorized the ABO incompatibility group into subgroups based on the number of TPE sessions: ≤3 sessions (5-year RFS: 76.2%, 5-year HCC recurrence: 17.4%), 4-5 sessions (5-year RFS: 68.5%, 5-year HCC recurrence: 16.5%), and ≥6 sessions (5-year RFS: 50.0%, 5-year HCC recurrence: 39.4%). Although these results were not statistically significant, a trend related to the number of TPE sessions was observed (P = 0.056 for RFS and P = 0.051 for HCC recurrence, Supplementary Figure S4).

In the multivariable Cox analyses (Table 3), the ABOi/TPE ≥6 group was significantly associated with RFS [hazard ratio (HR) = 1.99, 95% confidence interval (CI): 1.02–3.86, P = 0.042] and HCC recurrence (HR = 2.42, 95% CI: 1.05–5.57, P = 0.037).

**TABLE 3:** Multivariable Cox analysis for recurrence free survival and hepatocellular carcinoma recurrence.

Variables	Recurrence free survival HR (95% CI)	*P*	HCC recurrence^a^ HR (95% CI)	*P*
ABOi group				
ABOc	Reference		Reference	
ABOi/TPE ≤5	1.08 (0.63–1.85)	0.777	0.97 (0.46–2.01)	0.928
ABOi/TPE ≥6	1.99 (1.02–3.86)	0.042	2.42 (1.05–5.57)	0.037
Age, years	-	-	0.96 (0.92–1.00)	0.048
BMI	0.95 (0.89–1.01)	0.102	-	-
Pretransplant MELD	1.06 (1.03–1.09)	<0.001	-	-
Cold ischemic time, min	1.00 (1.00–1.01)	0.622	-	-
Transfusion RBC, pack	1.02 (1.01–1.04)	0.002	-	-
Log_AFP at LT	1.11 (0.98–1.25)	0.093	1.09 (0.94–1.25)	0.260
Log_PIVKA at LT	1.03 (0.90–1.18)	0.659	1.14 (0.98–1.34)	0.091
Pretransplant LRT, yes	2.91 (1.45–5.84)	0.003	7.00 (2.02–24.26)	0.002
Systemic treatment, yes	2.20 (1.37–3.53)	0.001	2.10 (1.16–3.82)	0.015
Viable tumor number	1.02 (1.01–1.04)	0.007	1.04 (1.01–1.07)	0.004
Maximum tumor size, cm	0.90 (0.82–0.98)	0.019	0.92 (0.82–1.03)	0.150
Microvascular invasion, yes	1.77 (0.97–3.22)	0.062	2.07 (1.01–4.24)	0.046
Poor differentiation, yes	1.30 (0.82–2.05)	0.268	1.69 (0.95–3.02)	0.076
Satellite nodule, yes	1.67 (0.90–3.10)	0.101	1.83 (0.92–3.63)	0.085
PVTT, yes	2.83 (0.98–8.16)	0.054	2.49 (0.57–10.86)	0.226

Variables which result p < 0.1 in univarible Cox analysis were included and represented at multivariable Cox analysis. Full univariate and multivariate results are represented at Supplementary Tables S2, S3.

^a^ Multivariable analysis for HCC recurrence was performed treating non-HCC death as competing risk.

ABOc, ABO compatible; ABOi, ABO incompatible; AFP, alpha-feto protein; BMI, body mass index; CI, confidence interval; HCC, hepatocellular carcinoma; HR, hazard ratio; LRT, locoregional treatment; MELD, model for end-stage liver disease; PIVKA, protein induced by vitamin K antagonist-II; PVTT, portal vein tumor thrombosis; TPE, therapeutic plasma exchange.

### Subgroup Analysis for HCC Recurrence

In the subgroup analysis (Table 4), the 5-year HCC recurrence rates were higher across all subgroups in the ABOi/TPE ≥6 group. Although this trend is numerically apparent, the small sample size limits the ability to confirm statistical significance. Interestingly, among patients with a tumor marker-based MoRAL score ≥100, the recurrence rate was significantly higher in the ABOi/TPE ≥6 group (56.7%) than in the ABOi/TPE ≤5 group with a MoRAL score ≥100 (16.1%, P = 0.017). However, patients with a MoRAL score <100 exhibited similar 5-year HCC recurrence rates between the two groups (17.6% vs. 20.5%, P = 0.75). Supplementary Figure S5 illustrates HCC recurrence based on the number of TPE sessions and the MoRAL score. As observed, a marked difference was evident between the ABOi/TPE ≥6 group with a high MoRAL score and the other groups (P = 0.0042).

**TABLE 4:** Subgroup analysis of 5-year hepatocellular carcinoma recurrence according to therapeutic plasma exchange numbers in ABO incompatible group.

Subgroups	Patient number		5 years HCC recurrence		*P*
	ABOi/TPE ≤5 (n = 75)	ABOi/TPE ≥6 (n = 30)	ABOi/TPE ≤5 (n = 75)	ABOi/TPE ≥6 (n = 30)	
Milan criteria					
Within	44	15	9.9%	35.2%	0.025
Above	31	15	27.2%	43.8%	0.267
Up-to-7					
Within	65	22	16.1%	35.5%	0.033
Above	10	8	27.1%	47.5%	0.569
French risk score					
≤2	56	20	14.8%	31.8%	0.082
>2	19	10	21.3%	55.0%	0.117
MoRAL score					
<100	49	16	17.6%	20.5%	0.750
≥100	26	14	16.1%	56.7%	0.017
IA titer at initial					
≤1:128	65	6	17.9%	50.0%	0.035
≥1:256	10	24	0.0%	64.1%	0.177
IA titer at LT					
≤1:16	61	11	19.4%	31.8%	0.287
≥1:32	14	19	0.0%	43.5%	0.026
IA titer rebound					
No	56	22	12.9%	28.6%	0.073
Yes	19	8	28.9%	66.7%	0.103
Post LT TPE					
No	61	19	12.1%	24.0%	0.203
Yes	14	11	37.3%	59.1%	0.246
Splenectomy					
No	71	23	17.9%	36.9%	0.075
Yes	4	7	0.0%	46.4%	0.137

ABOi, ABO incompatible; HCC, hepatocellular carcinoma; IA, isoagglutinin; LT, liver transplantation; MoRAL, model of recurrence after liver transplant; TPE, therapeutic plasma exchange.

Regarding the tumor burden criteria, the ABOi/TPE ≤5 group of patients within the Milan criteria exhibited a significantly lower 5-year HCC recurrence rate (9.9%) than the ABOi/TPE ≥6 group (35.2%, P = 0.025). Additionally, the 5-year HCC recurrence rate was significantly lower in the ABOi/TPE ≤5 group of patients within the Up-to-7 criteria (16.1%) than in the ABOi/TPE ≥6 group (35.5%, P = 0.033).

In the subgroup analysis based on immunological classification, patients with an initial IA titer ≤1:128 demonstrated a significantly higher recurrence rate in the ABOi/TPE ≥6 group (50.0%) than in the ABOi/TPE ≤5 group (17.9%, P = 0.035). However, patients with an IA titer ≥1:32 at LT had a significantly higher recurrence rate in the ABOi/TPE ≥6 group (43.5%) than in the ABOi/TPE ≤5 group (0.0%, P = 0.026).

## Discussion

The study evaluated the impact of pretransplant TPE sessions on HCC recurrence in patients undergoing ABOi LDLT and determined if limiting TPE sessions to fewer than six can enhance oncologic outcomes. We found that in the ABOi LDLT group, patients who underwent more than six pretransplant TPE sessions exhibited significantly worse HCC RFS and recurrence outcomes, with a similar trend observed in the subgroup analysis. Interestingly, the MoRAL score, which includes biomarkers, revealed that poorer oncologic outcomes were particularly pronounced in the high MoRAL score group. This suggests that in patients requiring a greater number of TPE sessions, biomarkers, in addition to tumor size, may play a crucial role in influencing HCC outcomes.

Moreover, the immunologic status at the time of transplantation is a critical factor influencing HCC recurrence [31, 32]. The need for multiple pretransplant TPE sessions may reflect an underlying immune dysregulation that could contribute to an increased risk of HCC recurrence. In particular, alterations in immune surveillance due to intensified desensitization protocols may affect the tumor microenvironment, potentially facilitating HCC recurrence [33, 34]. Similarly, ischemia-reperfusion injury (IRI) plays a crucial role in shaping the post-transplant microenvironment, influencing oncologic outcomes. Recent studies suggest that machine perfusion may help reduce HCC recurrence by mitigating IRI-induced inflammation and creating a more favorable post-transplant microenvironment [35–37]. Thus, assessing and managing the pretransplant immunologic status is essential for optimizing long-term oncologic outcomes in ABOi LDLT. A tailored approach that considers both desensitization requirements and immune profiling may help refine patient selection and improve posttransplant HCC prognosis. The strengths of our study include a well-organized dataset and a standardized desensitization protocol within the context of ABOi LDLT.

Globally, there has been a growing demand for LT as a definitive treatment for HCC, particularly for LDLT and ABOi LDLT due to organ shortages [15, 38]. In many countries outside East Asia, there is greater availability of deceased donors (DD), resulting in a predominant reliance on DDLT [38–40]. Consequently, these regions have limited cases and data regarding ABOi LDLT and the frequent use of TPE. In contrast, due to extreme shortages of deceased donors in Korea, LDLT is commonly performed for HCC [40, 41]. Paradoxically, this societal impact of deceased donor shortages has contributed to the accumulation of extensive data on ABOi LDLT, particularly in cases with high ABO antibody titers and a greater number of TPE sessions.

TPE is an intervention that involves the extracorporeal removal, return, or exchange of blood plasma or its components [42, 43]. The fundamental mechanism of this procedure is achieved through centrifugation or filtration using semipermeable membranes [44, 45]. In ABOi LDLT, the primary purpose of TPE is to remove IA. However, because this procedure is not selective, other immune-related factors in the blood are also removed, which presents a theoretical concern. Consequently, TPE is typically used as a primary or adjunctive treatment for conditions such as neurological diseases—including multiple sclerosis, amyotrophic lateral sclerosis, and myasthenia gravis—as well as autoimmune diseases like systemic lupus erythematosus and Kawasaki disease. Recent studies in this field have indicated that TPE promotes the differentiation and function of regulatory T cells [46–53].

Upon reviewing prior studies, it was noted that desensitization through pretransplant TPE or induction therapy in immunologically high-risk groups is associated with an increased cancer risk in certain malignancies (Table 5) [22, 23, 54, 55]. Although specific studies on ABOi LDLT are lacking, and the existing literature did not establish consistent protocols for TPE in kidney transplantation, direct comparisons with our study are challenging. Nevertheless, these findings underscore the relevance of desensitization and induction therapy concerning cancer risk, which was considered in our research.

**TABLE 5:** Previous studies regarding pretransplant desensitization and cancer risk.

Study	Yang, C.Y., et al.^a^	Motter, J.D., et al.^b^
Country	Taiwan	USA
Study period	2007–2013	1997–2016
Transplantation	Kidney	Kidney
Compared groups	DSA+ (n = 22) vs DSA – (n = 152)	ABOi LDKT (n = 858) vs ABOc LDKT (n = 12,239)
Plasmapheresis number	At least 4 cycles in DSA+ group	Not provided
Cancer type	Urothelial, endometrial, colon, and thyroid cancer	Colorectal cancer

*Cancer incidence*	DSA+ 19.6% vs DSA- 8.5% for 5 years (HR = 7.81, p = 0.028)	ABOi 0.6% vs ABOc 0.3% (HR = 3.27, p = 0.002)

*Hypothesis for higher cancer incidence*	Desensitization therapy for DSA+ including TPE might increase cancer	Desensitization therapy might increase cancer


^a^ Yang, C.Y., et al., *Renal transplantation across the donor-specific antibody barrier: Graft outcome and cancer risk after desensitization therapy*. J Formos Med Assoc, 2016. 115 (6): p. 426–33.

^b^ Motter, J.D., et al., *Cancer Risk Following HLA-Incompatible Living Donor Kidney Transplantation*. transplant direct, 2023. 9 (8): p. e1505.

ABOc, ABO compatible; ABOi, ABO incompatible; DSA, donor specific antibody; HR, hazard ratio; LDKT, living donor kidney transplantation; TPE, therapeutic plasma exchange.

Recent trends suggest that the outcomes of ABOi LDLT, including HCC outcomes and oncologic survival benefits, are comparable to those of ABOc LT [18, 19, 21]. However, these studies did not account for the cumulative and long-term effects of TPE, which prompted the initiation of our research.

In our study, the data indicated that ABOi patients requiring six or more pretransplant TPE sessions exhibited significantly poorer RFS and higher rates of HCC recurrence than ABOc patients. Additionally, our subgroup analysis shows that a higher number of pretransplant TPE sessions (≥6) was associated with a statistically significant increase in the 5-year HCC recurrence rate across several subgroups, including those within the Milan and Up-to-7 criteria, those with a high MoRAL score, and those with lower initial IA titers and higher IA titers at the time of LT.

Notably, within the size-based criteria, the ABOi/TPE ≥6 group exhibited a significantly higher recurrence rate. In contrast, regarding the tumor marker-based MoRAL score, a higher recurrence rate was observed in the ABOi/TPE ≥6 group only among patients with a score above 100. This suggests that among patients with a lower size-based tumor burden and a higher biologic-based tumor burden, those requiring more TPE sessions tended to experience poorer oncologic outcomes. Furthermore, this implies that the number of TPE sessions may be a more critical factor than the IA titer in influencing these outcomes.

Unlike previous studies, we focused on the immunomodulatory effects of T-regulatory (T-reg) cells induced by TPE and their association with HCC recurrence. As discussed earlier, while it is well established that T-reg cells are effective in treating autoimmune and neurological disorders, there are theoretical concerns that this process may reduce patient resistance to cancer [56, 57]. The literature indicates that the activation of T-reg cells can increase the risk of cancers such as HCC, with CD4^+^CD25+FoxP3+ T cells playing a significant role in this risk [58–60]. Although the exact cytokines and mechanisms through which these cells interact with others remain unclear, their differentiation within the tumor microenvironment (TME) has been observed [33, 34, 61], suggesting a potential increase in poor long-term cancer outcomes in various malignancies, including HCC. This information is illustrated in Supplementary Figure S6.

In summary, our hypothesis suggests that plasmapheresis induces the activation of T-reg cells, particularly CD4^+^ with CD25high, FoxP3+ effector T-reg cells, leading to an immunosuppressive effect within the tumor microenvironment that may facilitate tumor progression in various malignancies, including HCC. While some aspects of this pathway remain unexplained in the current foundational research, further studies are warranted to elucidate these mechanisms. Notably, the cumulative effect of TPE in the context of ABOi LT has not been extensively studied, underscoring the significance of our research.

This study has some limitations, including its retrospective and non-randomized design, the low number of ABOi/TPE ≥6 patients from a single center (n = 30), and the lack of fully established theoretical hypotheses or evidence to support our claims. Also, patients requiring more pretransplant TPE sessions may have additional unknown risk factors for HCC recurrence, highlighting the need for prospective studies to assess their impact on posttransplant outcomes [62]. However, despite these limitations, our study is significant, as it is the first to investigate the relationship between HCC outcomes and the number of preoperative TPE sessions and emphasizes the importance of comprehensive pretransplant evaluations in refining risk assessment for ABOi LDLT. In the future, we aim to address these limitations by increasing the sample size and establishing a more robust theoretical framework.

## Conclusion

This study demonstrated that the administration of more than six pretransplant TPE sessions in patients with HCC undergoing ABOi LDLT was associated with poorer oncologic outcomes. Based on our clinical findings and the theoretical association between TPE and HCC oncologic outcomes, we propose that limiting the number of TPE sessions to fewer than six may improve cancer outcomes in patients with HCC receiving ABOi LDLT. A strategy to reduce the number of TPE sessions to fewer than five should be implemented if possible when planning ABOi LDLT for HCC patients, ensuring adequate immunological stability through isoagglutinin titer control and maintaining comparable levels of immunological risk.

## Data Availability

The raw data supporting the conclusions of this article will be made available by the authors, without undue reservation.

## Footnotes

1
http://cran.r-project.org/

## Abbreviations

ABOc, ABO-compatible; ABOi, ABO-incompatible; AFP, Alpha-fetoprotein; aHR, Adjusted Hazards ratio; CD, Cluster of differentiation; CI, Confidence interval; DDLT, Deceased donor liver transplantation; MELD, Model for end-stage liver disease; HCC, Hepatocellular carcinoma; HR, Hazards ratio; IA, Isoagglutinin; LDLT, Living donor liver transplantation; LT, Liver transplantation; MoRAL, Model of Recurrence After Liver transplant; PIVKA-II, protein induced by vitamin K absence or antagonist II; PVTT, portal vein tumor thrombosis; RFS, Recurrence-free survival; TPE, Therapeutic plasma exchange.
