# Corrigendum: Number of Pretransplant Therapeutic Plasma Exchange Sessions Increase the Recurrence Risk of Hepatocellular Carcinoma in ABO-Incompatible Living Donor Liver Transplantation

**DOI:** 10.3389/ti.2025.15498

**Published:** 2025-09-17

**Authors:** Young Jin Yoo, Deok-Gie Kim, Eun-Ki Min, Seung Hyuk Yim, Mun Chae Choi, Hwa-Hee Koh, Minyu Kang, Jae Geun Lee, Myoung Soo Kim, Dong Jin Joo

**Affiliations:** ^1^ Department of Surgery, Research Institute for Transplantation, Yonsei University College of Medicine, Seoul, Republic of Korea; ^2^ Division of Transplant Surgery, Department of Surgery, Yongin Severance Hospital, Yonsei University College of Medicine, Seoul, Republic of Korea; ^3^ Department of Surgery, Graduate School of Medicine, Yonsei University College of Medicine, Seoul, Republic of Korea

**Keywords:** ABO-incompatible living donor liver transplantation, hepatocellular carcinoma, plasma exchange, surgical oncology, oncologic outcome

In the published article, there was an error regarding the corresponding authorship. The article omitted the designation of Deok-Gie Kim as a co-corresponding author.

The correct corresponding authors are listed below:

Corresponding author:

Dong Jin Joo,

Email: djjoo@yuhs.ac

Deok-Gie Kim,

Email: mppl01@yuhs.ac

The authors apologize for this error and state that this does not change the scientific conclusions of the article in any way. The original article has been updated.

