# Supplementary material for: Number of Pretransplant Therapeutic Plasma Exchange Sessions Increase the Recurrence Risk of Hepatocellular Carcinoma in ABO-Incompatible Living Donor Liver Transplantation
Source: Transpl Int. 2025 Aug 13;38:14304. doi: 10.3389/ti.2025.14304 (PMC12380628; doi:10.3389/ti.2025.14304)
Supplement: Supplementary file 1 [file DataSheet1.pdf]

SUPPLEMENTAL DIGITAL CONTENT

Capsule Sentence Summary

More than six pretransplant therapeutic plasma exchange(TPE) sessions can worsen HCC outcomes after ABOi LDLT. High TPE group showed lower recurrence-free survival and higher recurrence rates. Reducing TPE sessions while maintaining immunological stability through isoagglutinin titer control should be considered.

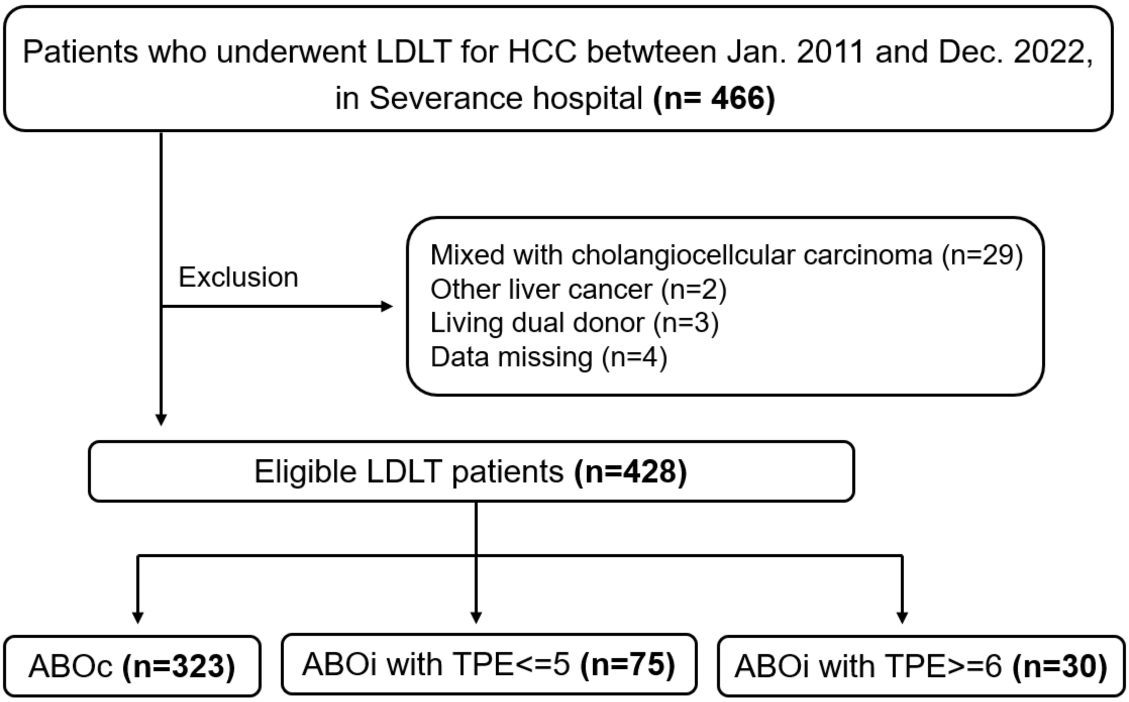

Figure S1. Study population

LDLT, living donor liver transplantation; HCC, hepatocellular carcinoma; ABOc, ABO-compatible; ABOi, ABO-incompatible; TPE, therapeutic plasma exchange.

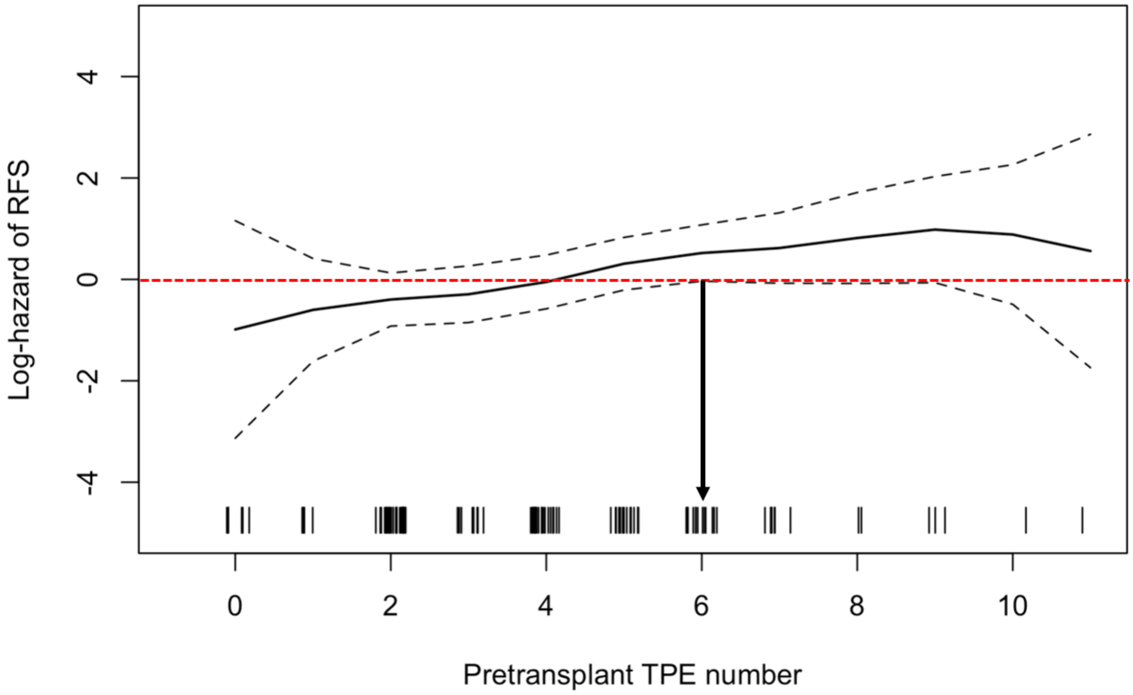

Figure S2. Cubic spline model for assessing proper pretransplant TPE number cutoff

RFS; recurrence free survival, TPE; plasma exchange.

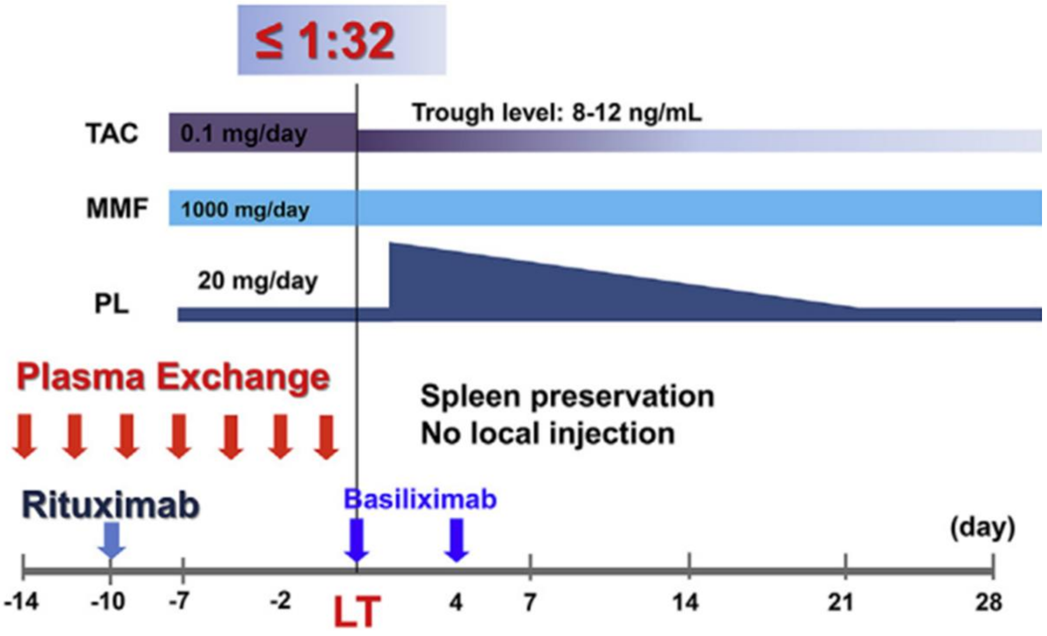

Figure S3-1. The modified desensitization protocol at YUHS for ABOi LDLT, since 2012

YUHS, Yonsei University Health System; ABOi, ABO incompatible; LDLT, living donor liver transplantation; TAC, tacrolimus; MMF, mycophenolate mofetil; PL, prednisolone; LT, liver transplantation.

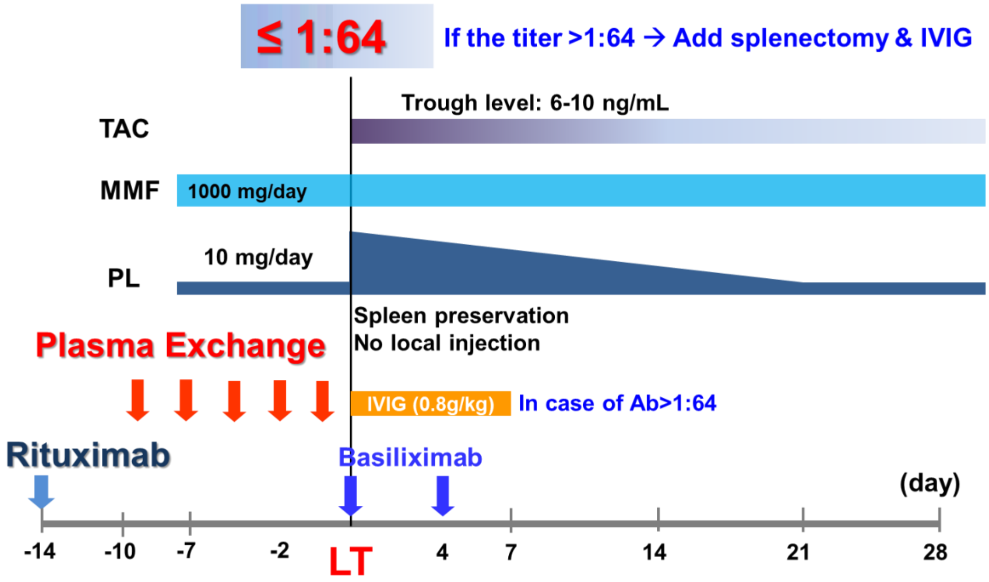

Figure S3-2. The modified desensitization protocol at YUHS for ABOi LDLT, since 2019

Pre-operative IVIG is considered when high titer (>=1:256) or titer is not effectively decreased after TPE (500mg/kg IVIG after TPE). Pre-operative TPE number is determined by anti-ABO IA titer (TPE*2 times <= 1:64, TPE*4 times in 1:128, TPE*6 times >= 1:256). Post-operative IVIG used when IA titer at LT is >=1:128 (800mg/kg IVIG for 1 week).

YUHS, Yonsei University Health System; ABOi, ABO incompatible; LDLT, living donor liver transplantation; IVIG, intravenous immunoglobulin; LT, liver transplantation; TAC, tacrolimus; MMF, mycophenolate mofetil; PL, prednisolone; PGE-1, prostaglandin E1; TPE, therapeutic plasma exchange; IA, isoagglutinin.

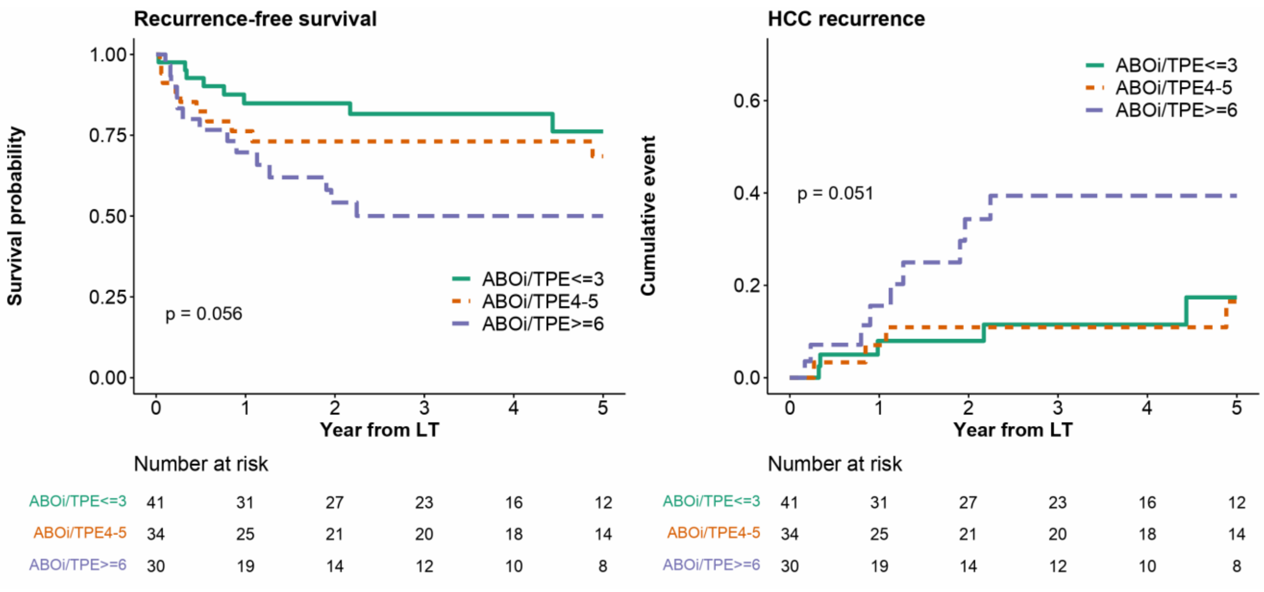

Figure S4. Kaplan-Meier curve of RFS and HCC recurrence in ABOi subgroup, according to plasma exchange numbers.

RFS, recurrence free survival; HCC, hepatocellular carcinoma; ABOi, ABO incompatible; TPE, therapeutic plasma exchange; LT, liver transplantation.

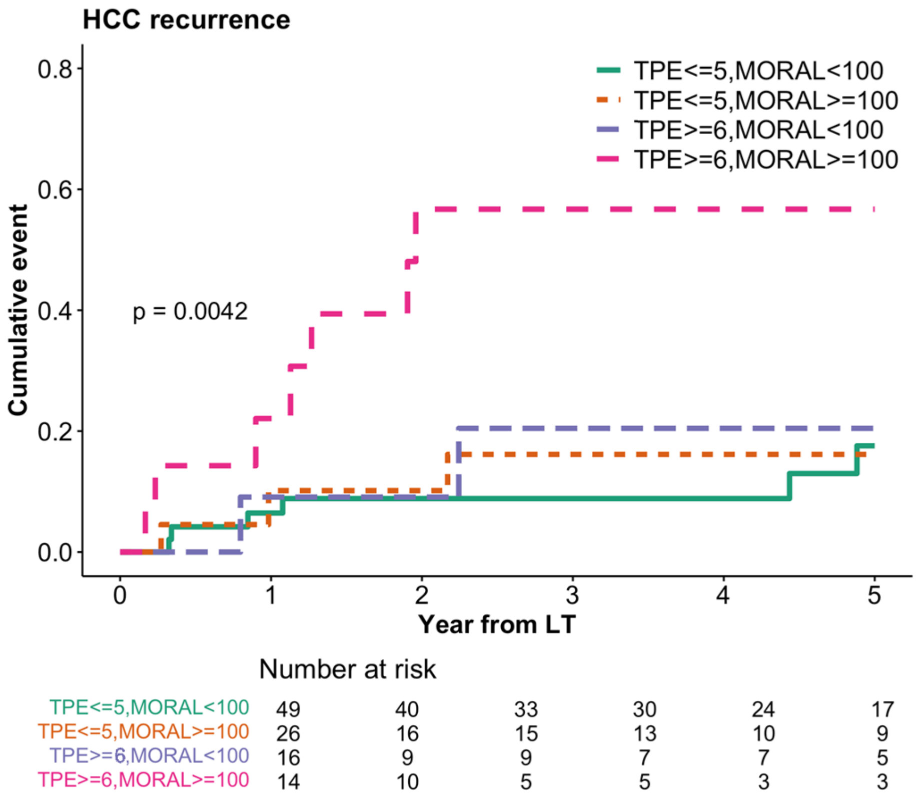

Figure S5. HCC recurrence in ABOi subgroup, according to plasma exchange numbers and MORAL score.

HCC, hepatocellular carcinoma; TPE, therapeutic plasma exchange; MORAL, Model Of Recurrence After Liver transplant; LT, liver transplantation.

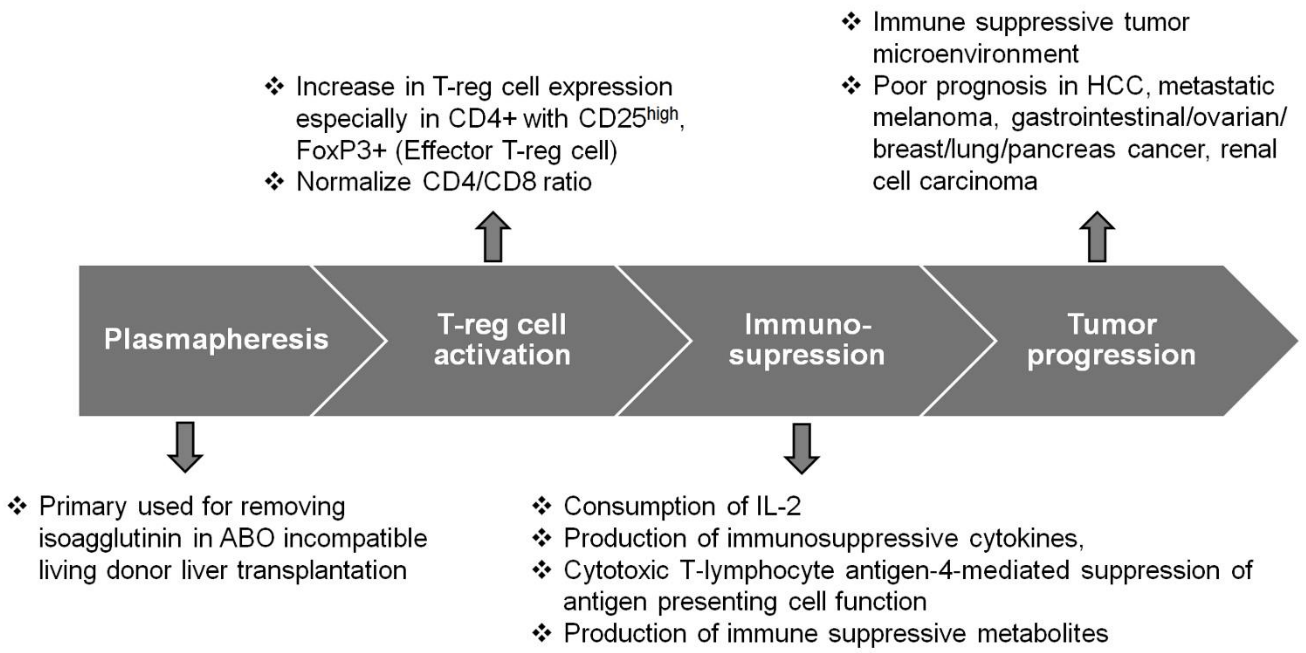

Figure S6. plasmapheresis and tumor progression flowchart

This diagram outlines the impact of TPE on T-reg cell activation and its subsequent effects on immunosuppression and tumor progression. TPE is primarily employed to remove isoagglutinin in ABO incompatible living donor liver transplantation, which increases T-reg cell expression, particularly effector T-reg cell. This immune modulation contributes to IL-2 consumption and enhances the production of immunosuppressive cytokines, resulting in a more immunosuppressive tumor microenvironment and poorer prognostic outcomes in HCC.

T-reg, T-regulatory; FoxP3, forkhead box P3; IL, interleukin; HCC, hepatocellular carcinoma.

Table S1. Univariate logistic regression analysis for 5 year HCC recurrence for recipient and donor ABO blood type, according to therapeutic plasma exchange number

	ABOi/TPE≤5 (n=75) HR (95% CI)	*P*†	ABOi/TPE≥6 (n=30) HR (95% CI)	*P*†
Recipient ABO type				
A	Reference			
B	2.67 (0.57 - 12.40)	0.211	1.00 (0 - Inf)	1.000
O	0.36 (0.04 - 3.48)	0.380	2.13 x 10^(7) (0 - Inf)	0.995
Donor ABO type				
A	Reference			
AB	0.41 (0.07 - 2.51)	0.336	4.31 x 10^(-8) (0 - Inf)	0.995
B	0.59 (0.12 - 2.98)	0.527	0.69 (0.13 - 3.61)	0.658
A to O	0.56 (0.06 - 4.93)	0.603	1.82 (0.36 - 9.27)	0.472

† Multivariate analysis was not performed due to the absence of variables with statistically significant p-values in the univariate analysis.

Table S2. Univariate and multivariate analysis of risk factors for recurrence free survival, according to ABOc/ABOi and TPE numbers.

Variables		Univariable HR (95% CI)	*P*	Multivariable HR (95% CI)	*P*
ABOi group	ABOc	Reference		Reference	
	ABOi/TPE≤5	1.13 (0.68-1.90)	0.633	1.08 (0.63-1.85)	0.777
	ABOi/TPE≥6	2.51 (1.42-4.46)	0.002	1.99 (1.02-3.86)	0.042
Age	Years	0.99 (0.96-1.01)	0.331	-	
Sex	Female	0.81 (0.48-1.36)	0.427	-	
BMI		0.93 (0.88-1.00)	0.037	0.95 (0.89-1.01)	0.102
LT year	*2011-2015*	Reference		Reference	
	2016-2019	1.25 (0.78-2.00)	0.363		
	2020-2022	1.53 (0.93-2.54)	0.095		
Underlying for HCC	*Hepatitis B*	Reference			
	Hepatitis C	0.84 (0.39-1.82)	0.659	-	
	Non-B, Non-C	0.54 (0.28-1.04)	0.067	-	
Hypertension	Yes	0.88 (0.55-1.41)	0.587	-	
Diabetes mellitus	Yes	0.94 (0.61-1.42)	0.756	-	
Pretransplant MELD		1.07 (1.04-1.10)	<0.001	1.06 (1.03-1.09)	<0.001
Donor age	Years	1.00 (0.99-1.02)	0.650	-	
Donor sex	Female	0.83 (0.56-1.25)	0.374	-	
GRWR†	< 0.8	1.59 (0.85-2.98)	0.145	-	
Macrovesicular steatosis	≥ 10%	0.58 (0.29-1.16)	0.122	-	
Cold ischemic time	Minutes	1.00 (1.00-1.01)	0.019	1.00 (1.00-1.01)	0.622
Transfusion RBC	Packs	1.03 (1.02-1.04)	<0.001	1.02 (1.01-1.04)	0.002
Log_AFP	at LT	1.24 (1.12-1.38)	<0.001	1.11 (0.98-1.25)	0.093
Log_PIVKA	at LT	1.35 (1.22-1.49)	<0.001	1.03 (0.90-1.18)	0.659
Hepatectomy history	Yes	1.37 (0.87-2.14)	0.173	-	
Pretransplant LRT	Yes	2.96 (1.58-5.54)	0.001	2.91 (1.45-5.84)	0.003
Systemic treatment	Yes	3.66 (2.40-5.59)	<0.001	2.20 (1.37-3.53)	0.001
Total necrosis	Yes	1.26 (0.78-2.03)	0.352	-	
Viable tumor number		1.04 (1.02-1.05)	<0.001	1.02 (1.01-1.04)	0.007
Maximum tumor size	cm	1.10 (1.05-1.17)	<0.001	0.90 (0.82-0.98)	0.019
Microvascular invasion	Yes	2.64 (1.79-3.90)	<0.001	1.77 (0.97-3.22)	0.062
Poor differentiation	Yes	2.25 (1.53-3.31)	<0.001	1.30 (0.82-2.05)	0.268
Satellite nodule	Yes	3.62 (2.34-5.60)	<0.001	1.67 (0.90-3.10)	0.101
PVTT	Yes	6.14 (2.67-14.12)	<0.001	2.83 (0.98-8.16)	0.054

† Graft weight was directly measured during operation.

ABOc, ABO compatible; ABOi, ABO incompatible; TPE, therapeutic plasma exchange; HR, hazard ratio; CI, confidence interval; BMI, body mass index; LT, liver transplantation; HCC, hepatocellular carcinoma; MELD, model for end-stage liver disease; GRWR, graft recipient weight ratio; AFP, alpha-feto protein; PIVKA, protein induced by vitamin K antagonist-II; LRT, locoregional treatment; PVTT, portal vein tumor thrombosis.

Table S3. Univariate and multivariate analysis of risk factors for HCC recurrence, according to ABOc/ABOi and TPE numbers.

**Variables**		**Univariable HR (95% CI)**	***P***	**Multivariable HR (95% CI)**	***P***
ABOi group	ABOc	Reference		Reference	
	ABOi/TPE≤5	0.90 (0.44-1.84)	0.770	0.97 (0.46-2.01)	0.928
	ABOi/TPE≥6	2.67 (1.31-5.46)	0.007	2.42 (1.05-5.57)	0.037
Age	Years	0.95 (0.92-0.98)	0.004	0.96 (0.92-1.00)	0.048
Sex	Female	0.58 (0.28-1.23)	0.155	-	
BMI		0.95 (0.88-1.03)	0.208	-	
LT year	*2011-2015*	Reference		Reference	
	2016-2019	1.01 (0.57-1.78)	0.972	-	
	2020-2022	0.99 (0.52-1.89)	0.972	-	
Underlying for HCC	*Hepatitis B*	Reference			
	Hepatitis C	0.56 (0.17-1.78)	0.326	-	
	Non-B, Non-C	0.42 (0.17-1.06)	0.066	-	
Hypertension	Yes	0.61 (0.31-1.20)	0.153	-	
Diabetes mellitus	Yes	0.62 (0.34-1.12)	0.116	-	
Pretransplant MELD		0.96 (0.90-1.02)	0.157	-	
Donor age	Years	0.98 (0.96-1.01)	0.196	-	
Donor sex	Female	0.84 (0.50-1.40)	0.507	-	
GRWR†	< 0.8	1.17 (0.47-2.92)	0.736	-	
Macrovesicular steatosis	≥ 10%	0.52 (0.21-1.29)	0.156	-	
Cold ischemic time	Minutes	1.00 (1.00-1.01)	0.390	-	
Transfusion RBC	Packs	1.02 (1.00-1.04)	0.039	-	
Log_AFP	at LT	1.38 (1.22-1.57)	<0.001	1.09 (0.94-1.25)	0.260
Log_PIVKA	at LT	1.43 (1.26-1.61)	<0.001	1.14 (0.98-1.34)	0.091
Hepatectomy history	Yes	1.57 (0.90-2.73)	0.113	-	
Pretransplant LRT	Yes	5.44 (1.98-14.98)	0.001	7.00 (2.02-24.26)	0.002
Systemic treatment	Yes	3.99 (2.33-6.84)	<0.001	2.10 (1.16-3.82)	0.015
Total necrosis	Yes	0.71 (0.34-1.49)	0.362	-	
Viable tumor number		1.05 (1.03-1.07)	<0.001	1.04 (1.01-1.07)	0.004
Maximum tumor size		1.14 (1.06-1.22)	<0.001	0.92 (0.82-1.03)	0.150
Microvascular invasion	Yes	4.85 (2.96-7.95)	<0.001	2.07 (1.01-4.24)	0.046
Poor differentiation	Yes	3.39 (2.06-5.58)	<0.001	1.69 (0.95-3.02)	0.076
Satellite nodule	Yes	6.55 (3.94-10.91)	<0.001	1.83 (0.92-3.63)	0.085
PVTT	Yes	6.31 (1.96-20.31)	0.002	2.49 (0.57-10.86)	0.226

† Graft weight was directly measured during operation.

HCC, hepatocellular carcinoma; ABOc, ABO compatible; ABOi, ABO incompatible; TPE, therapeutic plasma exchange; HR, hazard ratio; CI, confidence interval; BMI, body mass index; LT, liver transplantation; MELD, model for end-stage liver disease; GRWR, graft recipient weight ratio; AFP, alpha-feto protein; PIVKA, protein induced by vitamin K antagonist-II; LRT, locoregional treatment; PVTT, portal vein tumor thrombosis.
